# Prevalence of *Cryptosporidium*, *Blastocystis*, and other opportunistic infections in patients with primary and acquired immunodeficiency

**DOI:** 10.1007/s00436-018-5976-6

**Published:** 2018-06-26

**Authors:** Małgorzata Bednarska, Irena Jankowska, Andrzej Pawelas, Karolina Piwczyńska, Anna Bajer, Beata Wolska-Kuśnierz, Małgorzata Wielopolska, Renata Welc-Falęciak

**Affiliations:** 1 Department of Parasitology, Faculty of Biology, University of Warsaw, Warsaw, Poland; 2 Department of Gastroenterology, Hepatology, Nutritional Disorders and Pediatrics, Children’s Memorial Health Institute, Warsaw, Poland; 3 Department of Gastroenterology, Hepatology and Clinical Oncology, Medical Center for Postgraduate Education, Warsaw, Poland; 4 Immunology Clinic, Children’s Memorial Health Institute, Warsaw, Poland; 5 Department of Pediatry, Children’s Hospital, Otwock, Poland

**Keywords:** *Cryptosporidium* spp., *C. felis*, *Blastocystis hominis*, Opportunistic parasites, PID patients, Diarrhea

## Abstract

Intestinal opportunistic infections are often caused by unicellular parasites. Individuals with decreased immunity are particularly susceptible to infection by said microorganisms, and when they are infected, diarrhea can be the main clinical manifestation. However, intestinal parasites have rarely been taken into account in intestinal disorders. In our study, an investigation was conducted to determine the prevalence of intestinal micro-pathogens, such as *Cryptosporidium*, *Giardia*, *Blastocystis*, and microsporidia, in hospitalized patients with different immunological statuses. The study at hand indicates that protozoan parasitic infections are rare among immunodeficient patients in Poland. The overall prevalence of micro-pathogens among participants was 4.6%; it was three times higher in adults (12.5%) than in children (2.3%). *Cryptosporidium* and *Cyclospora* species (Apicomplexa) were diagnosed as the main cause of heavy diarrhea. Accordingly, adult patients were positive mainly for *Blastocystis* and microsporidia, while children were more often infected with the *Cryptosporidium* species.

## Introduction

Intestinal opportunistic infections are caused by viruses, bacteria, or unicellular parasites. Patients with impaired immunity are particularly susceptible to infections which may develop into severe illness. The first symptoms of intestinal parasitic infections are diarrhea and other intestinal disorders, such as cramping abdominal pains, nausea, vomiting, or low-grade fever. Diarrhea is ordinarily chronic and prolonged in the course of opportunistic diseases (Pierce and Kirkpatrick 2009; Nimri and Meqdam 2004). It can lead to dehydration (Bednarska et al. 2015), weight loss (Kucik et al. 2004), or even death (Cheng et al. 2005). *Cryptosporidium parvum* and *Cryptosporidium hominis* are prevailing microparasites in patients with immunodeficiency (Khan et al. 2017; Fayer 2010; Bajer et al. 2008). As of today, approximately 30 species and genotypic variants of *Cryptosporidium* have been described in mammals (Siński et al. 1998; Bajer et al. 2011), birds (Helmy et al. 2017), reptiles (Paiva et al. 2013), amphibian (Jirků et al. 2008), and fish (Ryan 2010). Most human diseases are provoked by *C. hominis* and *C. parvum* species which can infect more than 100 species of mammals (Caccio et al. 2005). Some less common species typical for animals, such as *Cryptosporidium meleagridis*, *Cryptosporidium felis*, *Cryptosporidium canis*, *Cryptosporidium muris*, and *Cryptosporidium suis*, have been reported mainly in humans with immunodeficiency (Cacciò et al. 2002; Wolska-Kusnierz et al. 2007; Bajer et al. 2008; Xiao 2010). It should be highlighted that *C. meleagridis*, previously described only in Turkey, has been noted in 1% of cryptosporidiosis in the UK (Mosier and Oberst 2000) and 10–20% in Peru (Cama et al. 2008).

Other opportunistic parasite species, such as microsporidia, *Cyclospora*, *Cystoisospora*, and *Blastocystis*, may also be associated with gastrointestinal diseases. It is currently unclear whether *Blastocystis* is a pathogen, a commensal, or an opportunistic organism. In favorable conditions, it causes intestinal disorders, but the infection may be either self-limiting or asymptomatic (Tan 2004; Scanlan and Stensvold 2013).

*Cyclospora* and *Cystoisospora* are most commonly associated with diarrhea in travelers, especially those visiting endemic areas (Legua and Seas 2013). Parasitic infections may cause a significant problem in immunocompromised persons (very young, elderly, after transplantation, and with AIDS) (Forrest 2004; Lewthwaite et al. 2005; Barsoum 2004). Transplant recipients are more likely to suffer from parasitic invasions as a consequence of immunosuppressive therapy. In general, gastrointestinal infections have been increasingly reported in this risk group. There are a few epidemiological studies carried out worldwide to examine the intestinal parasitic infections in liver or renal transplant recipients (Azami et al. 2010; Batista et al. 2011; Bednarska et al. 2013, 2014; Krause et al. 2012).

Microsporidia are a group of pathogens still poorly recognized and diagnosed in a human population. Of the 15 microsporidia species identified as human pathogens, two species cause gastrointestinal disease: *Enterocytozoon bieneusi* and *Encephalitozoon intestinalis*—the former being more commonly identified in solid-organ transplant recipients (Anane and Attouchi 2010).

In our study, the prevalence of intestinal micro-pathogens in hospitalized patients with different immunological statuses is defined. Furthermore, the pathogenicity detected in the patients with *Cryptosporidium* spp. and *Blastocystis hominis* is discussed.

## Materials and methods

### Stool samples

The study was carried out in three specialized hospitals in Warsaw along with its surrounding area during 2007–2015. Fecal samples were collected in hospital wards by medical practitioners for the purposes of routine bacteriological examinations and, subsequently, subjected to further retrospective examinations. Written informed consent was obtained from all patients, and the study protocol followed ethical guidelines of the 2013 Declaration of Helsinki. All ethical approvals for the study have been obtained according to Polish regulations. Fresh stool samples were obtained on two, three, or more occasions from patients and stored at + 4 °C. Samples were obtained from 285 patients (121 male and 164 female) with different immune statuses in the following departments: Children’s Memorial Health Institute in Warsaw (CMHI): (1) Gastroenterology, Hepatology, Nutritional Disorders and Pediatrics Clinic (CZW) (*n* = 147; 58M/89F); (2) the Immunology Clinic (CZD) (*n* = 34; 20M/14F); (3) Pediatric Department in General Hospital in Otwock (OT) (*n* = 40; 13M/27F); (4) Department of Gastroenterology, Hepatology and Clinical Oncology, Medical Center for Postgraduate Education (ON) (*n* = 64; 30M/34F). The patients were subdivided into two groups according to their age (221 under and 64 above 18 years old) and three groups based on their immunological status (Table 1).

**Table 1:** Characteristic of immunodeficiency degree use in this study. Number and distribution of the patients due to their immunological status

Degree of immunodeficiency (DOI)	Immunological status	Immune resistance decreasing factor	Number of patients
0	Immunocompetent	No	47
1	Mild immunodeficient	CVID, primary IgA immunodeficiency, chronic disease or transplant and 1–2 immunosuppressant drugs (low dose)	155
2	Heavy immunodeficient	PID, high doses of post-transplant or another medical immunosuppressant (2–3 drugs)	83

The first group involved 147 patients after liver transplantation under pharmacological immunosuppression [tacrolimus (TAC), sirolimus (SIR), cyclosporine (CSR) alone or collectively with steroids (ST), mycophenolate mofetil (MMF), or azathioprinum (AZP)]. The patients rarely manifested diarrhea or other intestinal disorders (6/147). The second group of children consisted of 34 patients, often with diarrhea (22/34), who presented impaired immunity due to confirmed (*n* = 32) or suspected (*n* = 2) primary immunodeficiencies (PID). The third group comprised 40 immunocompetent children with prolonged intestinal disorder (19/40) of unknown etiology. The fourth group consisted of 64 adult patients who presented acquired immunity disorders resulting from various acute diseases [Crohn’s disease (CD), colitis ulcerosa (UC), *Clostridium difficile* infection (CDI), Cytomegalovirus infections (CMV), rheumatoid arthritis (RAS), autoimmune enteropathy (AIE), hypereosinophilic syndrome (HES), common variable immunodeficiency (CVID), cholangiocarcinoma (CCC), unspecified immune resistance (UIR), radiotherapy (RTx)] and/or used drugs [glucocorticoids (GKS), AZP, 6-mercaptopurine (6-MP), MMF, Infliximabum (IFX)]. Most patients from this group presented prolonged diarrhea and/or other intestinal symptoms (54/64) often up to several months.

### Staining of fecal smears

Fecal smears were made from fresh stool specimens, which were air-dried, fixed in methanol, and stained with Ziehl-Neelsen (AquaMed, Poland) for *Cryptosporidium* spp. This method is highly effective in *Cyclospora cayetanensis* detection. The modified Weber’s chromotrope-based staining, i.e. trichrome staining (Chromotrope 2R Para-Pak Trichrome Stain, Meridian Diagnostics, Cincinnati, OH, USA) (Weber et al. 1992), was used for the *E. bieneusi* and *Encephalitozoon* spp. diagnoses. Smears were examined under oil immersion (× 1000 magnification). Indirect immunofluorescence assay (IFA) was performed for the verification or detection of *Cryptosporidium* and/or *Giardia* infections (Merifluor Cryptosporidium/Giardia kit, Meridian Diagnostics, USA) and diagnosed by direct immunofluorescence microscopy (× 400 magnification).

### PCR analysis

For DNA extraction, stool specimens were first concentrated by sedimentation (Bednarska et al. 2007). DNA extraction and purification were carried out using QIAamp DNA Stool Mini Kit (Qiagen), following the manufacturer’s protocol. Different sets of primers were used for PCR amplification with respect to the parasite species. A nested-PCR protocol was used to amplify the 18S rRNA gene fragments of *Cryptosporidium* spp. using primers previously described by Xiao et al. (1999). Additionally, a set of primers for Apicomplexa was used to confirm infection with *C. felis* (Herwaldt et al. 2003).

The next, “general” primers described by Raynaud et al. (1998) were used to amplify a 1200 bp conserved region of small-subunit ribosomal RNA genes (SSU-rDNA) with the aim of searching the range of human infecting microsporidial species, including *Encephalitozoon cuniculi*, *Encephalitozoon hellem*, *E. intestinalis*, and *E. bieneusi*. Species-specific primers were used to amplify a region of 545 bp from the SSU-rDNA of *E. intestinalis* (Valencáková et al. 2005), and species-specific primers were used to amplify a 607 bp fragment of the SSU-rDNA of *E. bieneusi* (da Silva et al. 1996).

*Blastocystis hominis* DNA was detected by PCR, previously described by Alfellani et al. (2013), to amplify the region of 600 bp from the SSU-rDNA.

Infection with *C. cayetanensis* was detected by microscopic methods and confirmed through nested PCR protocols used to amplify the 18S rRNA gene fragments using the published primer sets and thermal profiles. The nested PCR was performed to amplify a 500-bp fragment of *C. cayetanensis* 18S rDNA (Sulaiman et al. 2014).

All PCR products were subjected to electrophoresis in a 1.5% agarose gel stained with Midori Green stain (Nippon Genetics GmbH) and sequenced by a private company (Genomed S.A., Poland).

### Statistical analysis

SPSS 21 software was used for analysis. Patients presenting with diarrhea were compared with those without such symptoms. By the same token, adults and minors were compared.

Both the correlation between the degree of suppression and the occurrence of invasion, as well as the occurrence of diarrhea and the number of parasitic infections, were analyzed.

## Results

Out of the 283 patients (46 immunocompetent and 237 immunocompromised), a total of 5% (*n* = 14) were infected with intestinal parasites detected by microscopic, immunofluorescent, and/or PCR techniques. Additionally, three transplant recipients who were minors tested positive for *E. coli* bacteria strains which were closely related to enteroinvasive strains (99% homology) (Table 2).

**Table 2:** Microbiological and clinical features of patients with microparasitic infection

No.	PIC	Sex/age	Status immuno/(DI)	Transplant/another illness	Symptoms	Parasite species	Diagnosticmethods/ref.
1	9/04/CZD	M/4	PID, Hiper IgM (2)	Bone marrow	Prolonged diarrhea	*C. meleagridis*	Z-N, IFA, PCR (Wolska-Kusnierz et al. 2007)
2	17/05/CZD	M/5	PID, Sclerosis cholangitis, CD40 ligand deficiency (2)	Bone marrow	Prolonged diarrhea	*Cryptosporidium* sp.	Z-N, IFA, PCR (Wolska-Kusnierz et al. 2007)
3	35/07/CZD	F/2	ND (ND)	No	Diarrhea	*G. intestinalis*	IFA
4	204/CZW	M/3	PhI/.TAC, MMF (2)	Liver	No	*E. coli*	PCR
5	213/CZW	F/7	PhI/.TAC, MMF (1)	Liver	No	*E. coli*	PCR
6	220/CZW	F/6	PhI/TAC, MMF (1)	Liver	Diarrhea	*E. coli*	PCR
7	259/CZW	F/16	PhI/TAC, MMF (2)	Liver	No	*E. bieneusi* (JN107808)	Chr-2R, PCR
8	263/CZW	F/17	PhI/SIR, MMF (2)	Liver	No	*G. intestinalis*	IFA
9	348/CZW	F/9	PhI/SIR (2)	Liver	Diarrhea weight loss 1,5 kg	*C. felis* (KP675946)	Z-N, PCR IFA—neg!
10	707/ON	F/36	Full (1)	No/celiac disease	Prolonged diarrhea	*Enterocytozoon*/*Encephalitozoon*	Chr-2R, PCR (Bednarska et al. 2014)
11	709/ON	M/73	ImDef. (2)	No/diabetes rheumatoid arthritis	Prolonged diarrhea	*Enterocytozoon*/*Encephalitozoon*	Chr-2R, PCR (Bednarska et al. 2014)
12	718/ON	M/33	ImDef. (1)	No/IBDU	Prolonged diarrhea	*Enterocytozoon*/*Encephalitozoon*	Chr-2R, PCR (Bednarska et al. 2014)
13	757/ON	M/31	ImDef (2)	No/HIV+, lymphoma	Prolonged diarrhea	*C. parvum*	Z-N, IFA, PCR
14	758/ON	F/41	Full (0)	No/intestinal disorders	Weight loss	*B. hominis* Genotype ST-3	PCR
15	764/ON	M/23	Sterids (1)	No/colitis ulcerosa	Diarrhea/abdominal pain	*B. hominis* Genotype ST-2	PCR
16	PC1/ON	M/35	PhI/TAC (2)	Kidney	Diarrhea weight loss 15 kg	*C. cayetanensis* (KP642664)	Z-N, PCR (Bednarska et al. 2015)
17	PC2/ON	M/35	Full (0)	No	Defecation 3 times per day (no diarrhea)	*C. cayetanensis* (KP642665)/*B. hominis* (KP675947) Genotype ST-3	Z-N, PCR (Bednarska et al. 2015)

PIC, Patient Identification Code

PID, primary immunodeficiency

Chr-2R, smears stained by Chromotrope 2R method

ZN, smears stained by Ziehl-Neelsen method

IFA, MerIFluor *Cryptosporidium*/*Giardia* method

PCR, PCR with sequencing

TAC, tacrolimus

SIR, sirolimus

RA, rheumatoid arthritis

MMF, mycophenolate mofetil

MA, mycophenolic acid

DF, deflazacort

ImDef, other immunodeficiency (no transplant, no PID)

DI, degree of immunodeficiency

PhI, pharmacology immunodepression

The patients were infected with different *Cryptosporidium* species (1.4%, *n* = 4), *Giardia intestinalis* (0.7%, *n* = 2), *C. cayetanensis* (0.7%, *n* = 2), *B. hominis* (1%, *n* = 3), and presented with microsporidian invasion (*n* = 4). In one case, coinfection with *Cyclospora* and *Blastocystis* was detected (Table 2). The prevalence of pathogens was found in both immunocompetent (6.5%) and immunocompromised patients (4.6%). Micropathogen infections in children (< 18 years old, *n* = 221) and adults (> 18 years old, *n* = 62) were 3.2 and 12.9%, respectively (*p* = 0.226) (Table 3). There were significant differences in the prevalence of parasitic Protista (*Cryptosporidium*, *Giardia*, *Cyclospora*) between the male (5%) and female (0.6%) groups (*p* = 0.015, *df* = 1, *χ*^2^ = 5.885). The prevalence of Apicompexa infection with *Cryptosporidium* or *Cyclospora* species was significantly associated with diarrhea and heavy immunodeficient patients (*p* = 0.002, *df* = 2, *χ*^2^ = 12.88). There was an interesting link between micropathogen infections and immunosuppressed rates (*p* = 0.044, *df* = 2, *χ*^2^ = 6.242). Most parasitic infections were reported in patients with severe, second-stage immunodeficiency (6.1%), while in patients with mild or no immunosuppression, it was 0.6 and 2.2%, respectively.

**Table 3:** Prevalence of protozoan parasitic infections (*Cryptosporidium*, *Giardia*, *Blastocystis*, microsporidia) detected in patients of varying age and detection methods (PCR and microscopy)

Patients	% (total/infected)	% (children/infected)	% (adult/infected)
Parasites			
*Cryptosporidium* spp.	**1.4** (241/4)	**3.6** (221/3)	**1.6** (64/1)
*Giardia intestinalis*	**0.7** (241/2)	**0.9** (221/2)	**0** (64/0)
*Blastocystis hominis*	**1.2** (249/3)	**0** (187/0)	**4.7** (64/3)
Microsporidia *Enterocytozoon*/*Encephalitozoon*	**1.2** (209/4)	**0** (147/0)	**6.3** (64/4)
Total	**4.6** (285/13)	**2.3** (221/5)	**12.5** (64/8)

## *Cryptosporidium* infections

In our study, infections with *Cryptosporidium* occurred in four patients with diarrhea and heavy immunodeficiency. Among the four detected cases, three different species of *Cryptosporidium* were identified by PCR assay. Only one HIV+ adult patient (756/ON) was infected with *C. parvum*. Two prolonged infections in patients with PID (9/CZW, 17/CZW) were caused by *C. meleagridis* and *Cryptosporidium* spp., respectively (partially described by Wolska-Kusnierz et al. 2007). The infection caused by *C. felis* was detected in the liver transplant girl.

Our long-term study on two patients with prolonged cryptosporidiosis and heavy disorders was partially described by Wolska-Kusnierz et al. (2007). We reported the results of parasitological study, which was in progress for 7 years from 2007 to 2011–2014. Patient no. 9/04 infected with *C. meleagridis* underwent a four-time transplant in 2006 and, 6 years after transplantation with full immune reconstitution and no parasite infection, is alive and well. All results were obtained by three methods: ZN, IFA, and PCR—which at this time tested negative for *Cryptosporidium*.

Patient no. 17/05 with CD40 ligand deficiency complicated by cholangitis scleroticans and *Cryptosporidium* infection revealed *Cryptosporidium* infection at the age of 5, but long-term azithromycin treatment did not clear up or treat the infection. At age 7, he received matched unrelated stem cell transplantation. Liver failure with vanishing bile duct syndrome in the course of severe graft versus host disease (GVHD) occurred after transplantation. In March 2008, liver transplantation from an unrelated donor was successfully performed. In a follow-up study, we observed the clearance of *Cryptosporidium* infection together with full immune reconstitution. No recurrence of parasite infection was detected during the following 7 years of observation.

## *Blastocystis hominis* infection

A partially retrospective study regarding *B. hominis* was carried out on 249 patients (187 minors). Positive PCR results were obtained only from adult patients (4.7%, 3/64), two of which were hospitalized. The 23-year-old male suffered from *colitis ulcerosa*, infections with *Cytomegalovirus* (*CMV*), and *Clostridium difficile*. The 41-year-old female complained of intestinal disorders, such as abdominal pain, weight loss, and alternating rhythm of bowel movements. The third patient (PC2), infected with *Blastocystis*, was first diagnosed with *C. cayetanensis.* Coinfection with *Blastocystis* was detected based on the molecular study. Three defecations per day were reported as a physiological norm by this immunocompetent male (Bednarska et al. 2015).

**Fig. 1:**
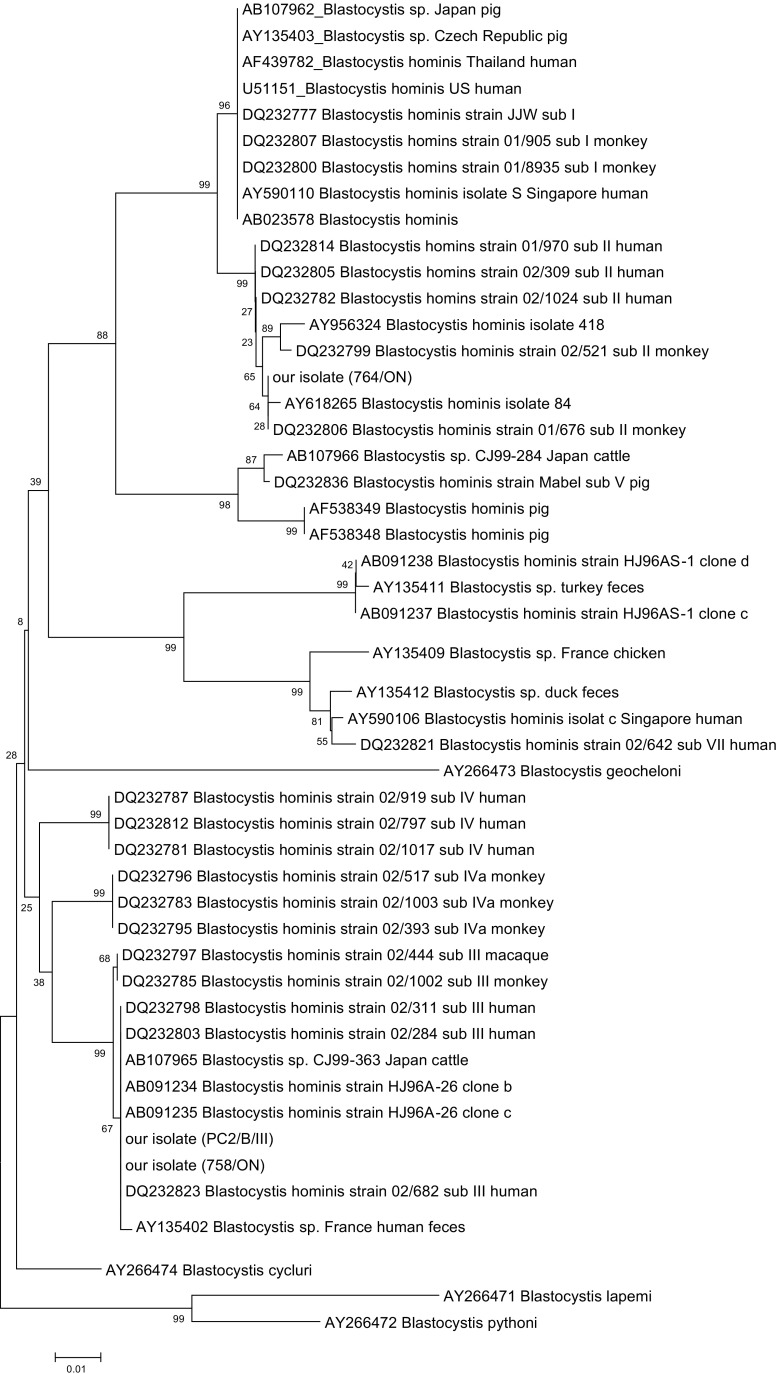
Evolutionary relationships of taxa. The evolutionary history was inferred using the Neighbor-Joining method. The percentage of replicate trees in which the associated taxa clustered together in the bootstrap test (1000replicates) is shown next to the branches. The tree is drawn to scale, with branch lengths in the same units as those of the evolutionary distances used to infer the phylogenetic tree. The evolutionary distances were computed using the Kimura2 parameter method and are in the units of the number of base substitutions per site. The analysis involved 51 nucleotide sequences. All positions containing gaps and missing data were eliminated. There were a total of 398 positions in the final data set. Evolutionary analyses were conducted in MEGA6

DNA sequence alignments and phylogenetic analysis were conducted using MEGA version 6.0. Two isolates from 758/ON and PC2/ON patients were closely related (identical), and both were grouped in the region (III) closely related to human isolates. The nucleotide sequences of the ITS fragment of DNA isolated from patient no. 764/ON were also related to genotypes rarely isolated from human specimens (region II) (Abe 2004). The relatedness of isolates, grouped by its sequence identity, is showed in the phylogenetic tree (Fig. 1). The GenBank accession numbers assigned to the sequences determined in this study are as follows: genotype 758/ON, MG905018, genotype 764/ON, MG905016, and genotype PC2/ON, MG905017.

## Discussion

### Prevalence of micro-parasitic infection

Microparasites, such as viruses and bacteria, may cause infective diarrhea in immunodeficient and post-transplant patients in endemic areas, yet the data regarding such infections in Poland are scarce (Table 4). The research presented here represents one of few studies of the incidence, disease manifestation, management, and outcome of microparasitic infection in transplant recipients and immunocompromised individuals. This study indicates that protozoan parasitic infections are rare among immunodeficient patients in Poland. The overall prevalence of micro-pathogens in the study participants was 4.6%, and it was three times higher in adults (12.5%) than in children (2.3%), which attests to the fact that microparasitic infections are the most frequent in patients who are not minors. Accordingly, adult patients tested positive mainly for *Blastocystis* and microsporidia, while children were more often infected with the *Cryptosporidium* species. It is worthy of attention that *Cryptosporidium* parasites, together with *G. intestinalis*, were included in the WHO’s “Neglected disease initiative” in 2004 (Savioli et al. 2006) due to their significance in public health.

**Table 4:** Prevalence of *Cryptosporidium* spp., *Giardia intestinalis*, *Blastocystis hominis*, microsporidia *Enterocytozoon*/*Encephalitozoon* in human using different (microscopic/IFA/PCR) methods in Poland, 2000–2017

Parasite species	N positive/N total	Immunological/medical status	Study period	Diagnostic method	References
*Cryptosporidium spp.*	9/221	Immunocompetent with diarrhea	2013–2017	LM	Kłudkowska et al. (2017)
	36/246	Immunocompetent with diarrhea	2006	LM (ZN) IFA, PCR	Rożej et al. (2010)
	1/35	PIDs	2002–2007		Bajer et al. (2008); Wolska-Kusnierz et al. (2007)
*Cryptosporidium parvum*	9/35	Immunocompetent/PIDs			
*C. hominis*	1/35	PIDs			
*C. meleagridis*	1/35	PIDs			
	1	HIV	nd	LM (ZN), PCR	Wesołowska et al. (2016)
*Cryptosporidium felis*	1	Liver transplant	2014	LM (ZN), PCR	This paper
*Giardia intestinalis*	3/232	Immunocompetent	nd	LM(DS, TS) PCR	Solarczyk et al. (2010)
	2/285	Immunocompetent/transplant recipient	2007–2016	IFA	This paper
	6/913	Immunocompetent	2008–2010	LM (DS)	Duda et al. (2015)
*Cyclospora cayetanensis*	2/2	Renal transplant/immunocompetent	2015	LM (ZN), PCR	Bednarska et al. (2015)
	3/221	Immunocompetent with diarrhea	2013–2017	LM	Kłudkowska et al. (2017)
*Blastocystis hominis*	3/249	Immunocompetent/transplant recipient/medical suppression	2007–2016	PCR	This paper
	140/913	Immunocompetent	2008–2010	LM (DS)	Duda et al. (2015)
*Enterocytozoon bieneusi*	1/60	Liver transplant recipient	2011	PCR	Bednarska et al. (2013)
*Enterocytozoon*/*Encephalitozoon*	10/80	Immunocompetent/PIDs/transplant recipient	2002–2008	LM (TS), PCR	Bednarska et al. (2014)
*Enterocytozoon bieneusi*	7/86	Renal transplant recipients	2013–2015	MS, PCR	Kicia et al. (2014)
*Encephalitozoon cuniculi*	15/86				

LM, light microscopy; ZN, Ziehl-Neelsen stain; JM, culture on Jones medium; DS, direct smear; TS, trichrome stain

### Detection and characterization of *Cryptosporidium* spp. and *Blastocystis* spp. isolates

It is known from earlier studies that cryptosporidiosis prevalence varies from 1 to 5% in children from developed countries and 50% in children from developing countries. Cryptosporidiosis can be found more often in young children and immunocompromised patients (especially those with HIV-associated immunosuppression) than in a healthy adult population (Cheng et al. 2005).

In our study, the *Cryptosporidium* species was detected in 3.6% of children, which is similar to the 1.2% in the Teheran study (Tahvildar-Biderouni and Salehi 2014), 2% in Poland (Solarczyk et al. 2010) 3.9% of the *Cryptosporidium*-infected children in the study of Tanzania (Tellevik et al. 2015), 4.6% in Ethiopia (de Lucio et al. 2016), and 2.4% Iranian children (Taghipour et al. 2011). *Cryptosporidium* prevalence found in this study is lower than that reported for children with diarrhea in Canada which was at 15.7% (Iqbal et al. 2015), 10.4% in Tanzania, and 15.1% in Qatar (Boughattas et al. 2017). These variations could be explained by the differences in the region of study, the hygiene practices, as well as the socio-economic status of participants involved in the studies.

By using molecular methods in this study, *C. parvum*, *C. meleagridis*, and *C. felis* were identified in diarrheic patients. All identified *Cryptosporidium* isolates are considered as zoonotic species which are commonly reported in humans and wildlife worldwide (Xiao 2010).

The distribution of *Cryptosporidium* species in humans varies across geographic areas and socioeconomic conditions (Chalmers and Katzer 2013). In European countries, the detection of infection with *C. parvum*, *C. hominis*, and *C. meleagridis* in humans increased. Infections with *C. canis* and *C. felis* are reported in studies conducted in developing countries (Xiao 2010).

In our study, two cases of chronic cryptosporidiosis (*C. meleagridis* and *Cryptosporidium* sp.) were reported among PID children. Hyper-IgM patients with *C. meleagridis* infection (partially reported by Wolska-Kusnierz et al. 2007) were monitored after four bone marrow transplantations (the last one performed in 2006), and the problem with cryptosporidiosis was resolved. Within a few years, no relapse to *Cryptosporidium* infection was observed. The resolution of opportunistic infections in immune-suppressed patients requires the restoration of mucosal immunity, usually achieved following the discontinuation of immunosuppressive drugs (Nachbaur et al. 1997). The patient diagnosed with CD40 ligand deficiency, sclerosis cholangitis, and prolonged cryptosporidiosis required bone marrow transplant as well as liver transplant after GVHD intended for the full immune reconstruction. Chronic cryptosporidiosis can cause GVHD after stem cell transplantation, thus requiring a reduction of immunosuppressive drugs and a specific therapy, whereas GVHD requires intensification of immunosuppression (Legrand et al. 2011; Washington and Jagasia 2009). Presumably, in the case in question, the heavy immunosuppression led to re-development of cryptosporidiosis. Relapse to cryptosporidiosis at this stage of treatment can be dangerous for the health and life of a patient. Therefore, control and prompt diagnosis of intestinal cryptosporidiosis are recommended.

*Cryptosporidium felis* has a visibly more restricted host range than *C. parvum* and, using molecular techniques, it has been confirmed that it may infect cats (Lucio-Forster et al. 2010), immunocompetent and immunocompromised humans (Cacciò et al. 2002), as well as cattle (Bornay-Llinares et al. 1999). Infection with *C. felis* was detected in a young female who had undergone a liver transplant. Importantly, this patient resided in a rural environment with direct access to dogs, cats, and other farm animals. The most probable source of infection comprised cats from the close surroundings. In children from developing countries, *C. felis* is responsible for as much as 3.3% of all cryptosporidiosis cases (Lucio-Forster et al. 2010).

*Cryptosporidium* infections were detected aided by microscopic studies, using Ziehl-Neelsen staining and the IFA method, while the species identification was confirmed using PCR. Interestingly, in this case, *C. felis* infection was not detected in immunofluorescent testing dedicated to detection of a wide range of *Cryptosporidium* species in stool specimens. These results suggest that studies on the transmission of zoonotic species are difficult due to the lack of suitable subtyping tools for the distinction of *Cryptosporidium* spp. (Ryan et al. 2014). Given the above, it is necessary that two different methods for the detection of *Cryptosporidium* and other parasitic infections in humans are employed. A diagnosis based only on a microscopic or immunofluorescent test or molecular methods only may lead to false-negative results. A molecular study is necessary to recognize the genotype and subtype of *Cryptosporidium* and to identify the organism responsible for infection along with the source and routes of transmission.

Infection with *Blastocystis* has been reported as asymptomatic, acute, or chronic symptomatic (Windsor et al. 2002; Tan 2004). This wide range of responses to infection could be related to genetic diversity. *Blastocystis hominis* was detected only in adult patients (4.7%), and in one male patient, co-infection with *C. cayetanensis* was found. In this study, the pathogen was detected only by molecular methods. An earlier study of patients with hematopoietic and lymphoid hyperplastic diseases (not published) compared light microscopy, immunofluorescence, ELISA, cultivation using Joni’s medium, and PCR as methods used for detection of *Blastocystis* sp. The most useful diagnostic methods seem to be cultivation (15%, 6/40) and PCR (17.5%, 7/40). Parasites were detected in 30% of patients using both above-mentioned methods. These results confirm that two diagnostic methods should be used in parasitological diagnostic.

The sources of infection with *Blastocystis* can be water or zoonotic transmission (Abe 2004), while the risk factors include immunocompromised health (Rao et al. 2003) or poor hygiene practices (Nimri and Meqdam 2004; Tan 2004). Pathogenicity of *Blastocystis* is controversial and still undefined. Thus, further research should be carried out to determine the potential risk associated with the invasiveness of their subtypes. The most dominant subtypes in humans are subtype 3 (41.7 to 92.3%) and subtype 1 (7.7 to 25%), followed by either subtype 6 (10 to 22.9%), subtype 2 (1.3 to 32.1%), or subtype 4 (1.3 to 37.5%)—the occurrence of which is associated with geographical distributions. In most studies, other genotypes (ST5, ST7–9) were identified at lower frequencies globally (Tan 2004; Alfellani et al. 2013). In our study, subtype 2 was detected in immunodeficient patients with *colitis ulcerosa* and co-infections with CMV and *C. difficile*. Subtype ST-3 was diagnosed in two immunocompetent patients, one of which was hospitalized due to weight loss and alternating rhythm of bowel movements. The other one was diagnosed with co-invasion during the prolonged, asymptomatic infection with *C. cayetanensis* (Bednarska et al. 2015). It is difficult to assess the impact of *B. hominis* infection on the chronic *C. cayetanensis* infection. The higher frequency of defecations in this patient could have been a symptom of irritable bowel caused by *Blastocystis*. Further research must be conducted to clarify the effects of *B. hominis* on intestinal peristalsis, asymptomatic *C. cayetanensis* infection, or any different microparasitic infection which may result in prolonged contamination of the environment by asymptomatic, but chronically infected patients.

### Diarrhea in immunodeficiency

A majority of diarrhea cases were due to non-parasitic infections. In total, 35% patients had symptoms at the time of the survey. Gastrointestinal symptoms were more often reported in children with PID (65%) and adult patients (84%). Post-transplant diarrhea is a common and distressing occurrence in patients, which can have significant deleterious effects on the clinical course and well-being of organ recipients. The true incidence of diarrhea in liver transplant recipients is unknown but possibly ranges from 10 to 43%—according to published studies in other solid organ and bone marrow transplantation (Azami et al. 2010; Galván et al. 2011; Agholi et al. 2013). Our observations did not agree with these data. In our study on liver transplant recipients, only 2% were infected with parasites, but diarrhea was occasionally presented (4%). Diarrhea could be a frequent side effect of immunosuppressive medications (mycophenolate mofetil (MMF), cyclosporine A (CSA), tacrolimus, and sirolimus) or an additional infectious agent, including viruses (e.g. *Cytomegalovirus*), bacteria, or fungi (e.g. *C. difficile*) (Bonatti et al. 2012; Song et al. 2006; Dave et al. 2014). More than half of the patients tested in this study had heavy immunodeficiency due to medications or diseases such as inflammatory bowel disease (e.g., *colitis ulcerosa*) or celiac disease, and probably these factors were the main reasons for intestinal disorders.

In conclusion, the current study illustrates the need to maintain a high index of suspicion for microparasites, especially *Cryptosporidium*, microsporidia, and *Blastocystis* in immunodeficient or transplant patients who present prolonged diarrhea. We diagnosed *Cryptosporidium* and *Cyclospora* species (both Apicomplexa) as the main cause of heavy parasitic diarrhea. In our opinion, parasitic infections should be diagnosed with two different methods for an accurate diagnosis. Probably, the routine stool evaluations for parasites may not identify rare zoonotic species or low intensity of parasites. Furthermore, our results imply that a molecular analysis used to identify the parasite species should be performed as soon as the zoonotic *Cryptosporidium* infection is suspected. Various other etiologies, including inflammatory bowel disease, must be considered in the differential diagnosis. This will allow choosing the proper treatment for specific parasite infections.
